# Salts of Antifolate Pyrimethamine with Isomeric Aminobenzoic Acids: Exploring Packing Interactions and Pre-Crystallization Aggregation

**DOI:** 10.3390/ijms27010180

**Published:** 2025-12-23

**Authors:** Karolina Cichocka, Magdalena Zimnicka, Karolina Kędra, Arkadiusz Gajek, Magdalena Ceborska

**Affiliations:** 1 Faculty of Mathematics and Natural Sciences, Cardinal Stefan Wyszyński University, 01-938 Warsaw, Poland; karolina.cichocka@student.uksw.edu.pl; 2 Institute of Organic Chemistry, Polish Academy of Sciences, 01-224 Warsaw, Poland; magdalena.zimnicka@icho.edu.pl; 3 Institute of Physical Chemistry, Polish Academy of Sciences, 01-224 Warsaw, Poland; kkedra@ichf.edu.pl (K.K.); agajek@ichf.edu.pl (A.G.)

**Keywords:** crystal engineering, co-crystallization, pyrimethamine, aminobenzoic acids, physicochemical properties

## Abstract

Pyrimethamine (PYR), a drug approved for the treatment of infections caused by protozoan parasites, is a multifunctional API based on 2,4-diaminopyrimidine scaffold. The present study aims toward the development of novel solid forms of PYR, by combining it with three isomeric aminobenzoic acids—2-aminobenzoic acid (2NH_2_-BA), 3-aminobenzoic acid (3NH_2_-BA), and 4-aminobenzoic acid (4NH_2_-BA). Solution crystallization led to the formation of three new solvated salts of PYR (PYR/2NH_2_-BA/EtOH/H_2_O, PYR/3NH_2_-BA/EtOH, and PYR/4NH_2_-BA/EtOH/H_2_O). The detailed physicochemical properties of the formed compounds were characterized by single-crystal X-ray diffraction (SC-XRD), FTIR, PXRD, thermogravimetry (TG), and differential scanning calorimetry (DSC). Additionally, the pre-crystallization solutions of PYR with 2NH_2_-BA, 3NH_2_-BA, and 4NH_2_-BA were studied by electrospray ionization mass spectrometry technique (ESI-MS), which enabled the observation of peaks corresponding to noncovalently bonded molecules, providing insight into their specific aggregation in a solution/gas phase environment. We identified different non-covalent aggregates, including self-aggregates of aminobenzoic acids and PYR/aminobenzoic acid associates of different stoichiometries.

## 1. Introduction

**Figure 1:**
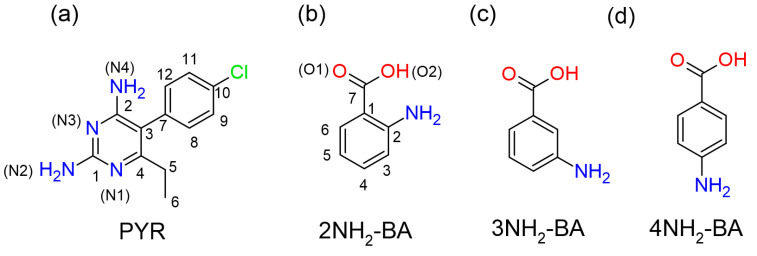
(**a**) Pyrimethamine (**b**) 2NH_2_-BA (**c**) 3NH_2_-BA (**d**) 4NH_2_-BA, with atom numbering.

Pyrimethamine (PYR, Figure 1a) [1] is a drug approved for the treatment of infections caused by protozoan parasites (e.g., malaria and toxoplasmosis). It acts by targeting plasmodium dihydrofolate reductase pDHFR, an essential enzyme in the synthesis of folic acid, which is required for DNA and RNA synthesis [2,3,4]. Recently, PYR [5], as well as its analogues [6,7,8,9] were found to show some anticancer activity in the number of in vivo model systems, arousing scientific interest in their possible new applications and in the improvement of their physiochemical properties. Such improvements may be possible by the formation of the salts or cocrystals of an API [10,11,12]. Currently, pyrimethamine salts and cocrystals with various carboxylic acids have been reported [13,14,15,16,17,18,19,20,21,22,23,24].

In our previous research we focused on the formation and characterization of pyrimethamine salts with isomeric monohydroxybenzoic acids (MHBAs), including 2-hydroxybenzoic acid (2OH-BA), 3-hydroxybenzoic acid (3OH-BA), and 4-hydroxybenzoic acid (4OH-BA), as well as with methyl-substituted 2-hydroxybenzoic acids: 3-methylsalicylic acid (3Me-SA), 4-methylsalicylic acid (4Me-SA), and 5-methylsalicylic acid (5Me-SA). In all of the studied cases (apart from cocrystallization with 4OH-BA, where we could not obtain crystals of the associate of any kind), N(1) nitrogen atom of PYR underwent protonation, while the carboxylic group of the acid molecule was deprotonated, resulting in the formation of the salt. Although all of the salts were obtained in the same experimental conditions (slow evaporation of 1:1 ethanolic solutions) the outcome differed, as in two cases (PYR/3Me-SA and PYR/5Me-SA) the product crystallized as an anhydrous salt, in one (PYR/3OH-BA) as a hydrated salt, and in the other two as an ethanol-solvated hydrated salt (PYR/2OH-BA and PYR/4Me-SA). The different substitutions of benzoic rings in the studied carboxylic acids did not change the main reaction outcome, but it did influence the overall 3D structure due to the different layout of hydrogen bonds. Similar observations were made by Muthiah et al. [24], who studied the formation of pyrimethamine salts with isomeric nitrobenzoic acids: 2-nitrobenzoic acid (2NO_2_-BA), 3-nitrobenzoic acid (3NO_2_-BA), and 4-nitrobenzoic acid (4NO_2_-BA). These results inspired us to focus our research on the study of how the change of the hydroxyl group into an amino group attached to the aromatic scaffold would change the possible outcome of the crystallization of substituted benzoic acids with pyrimethamine and how differences in the position of the amino substituent influence the 3D structure, and, subsequently, the properties of the obtained compounds. For our studies we chose 2-aminobenzoic acid (2NH_2_-BA, Figure 1b), 3-aminobenzoic acid (3NH_2_-BA, Figure 1c), and 4-aminobenzoic acid (4NH_2_-BA, Figure 1d), which are frequently used as coformers in crystallization with basic compounds [25,26,27,28,29,30,31,32,33,34,35,36,37,38,39,40,41,42,43,44,45,46].

In this work, three new pyrimethamine salts with isomeric aminobenzoic acids with 1:1 stoichiometric ratio were synthesized by solution crystallization. The detailed physicochemical properties of the formed compounds were characterized by single-crystal X-ray diffraction (SC-XRD), and thermal analysis (thermogravimetry (TG), and differential scanning calorimetry (DSC)). Additionally, we studied the pre-crystallization solutions of PYR and 2NH_2_-BA, 3NH_2_-BA, and 4NH_2_-BA using the mass spectrometry technique (electrospray ionization (ESI-MS)). Application of soft ionization techniques, such as ESI, enables the observation of peaks corresponding to noncovalently bonded molecules, providing insight into their specific aggregation in solution/gas phase environment [47,48]. We identified different non-covalent aggregates, including self-aggregates of aminobenzoic acids and PYR/aminobenzoic acids of different stoichiometries.

## 2. Results and Discussion

Within this work, three novel molecular salts of pyrimethamine with three isomeric aminobenzoic acids were successfully prepared by traditional solution method. All three obtained crystals were subjected to SC-XRD measurements and analysis. The obtained associates crystalized in the form of solvated salts—PYR/2NH_2_-BA/EtOH/H_2_O; PYR/3NH_2_-BA/EtOH, and PYR/4NH_2_-BA/H_2_O. In all three obtained crystal structures, hydrogen atoms protonating N(1) of PYR were visible on the Fourier difference maps. Additionally, analysis of the C−O bond lengths in the deprotonated carboxyl groups of PYR/NH_2_-BA associates was performed. As can be seen from Table 1, all C–O bond length values lie between the values for pure single (1.43 Å) and double bonds (1.23 Å) and are characteristic for the delocalized carboxylate anion (1.27 Å) [49].

### 2.1. SCXRD

**Figure 2:**
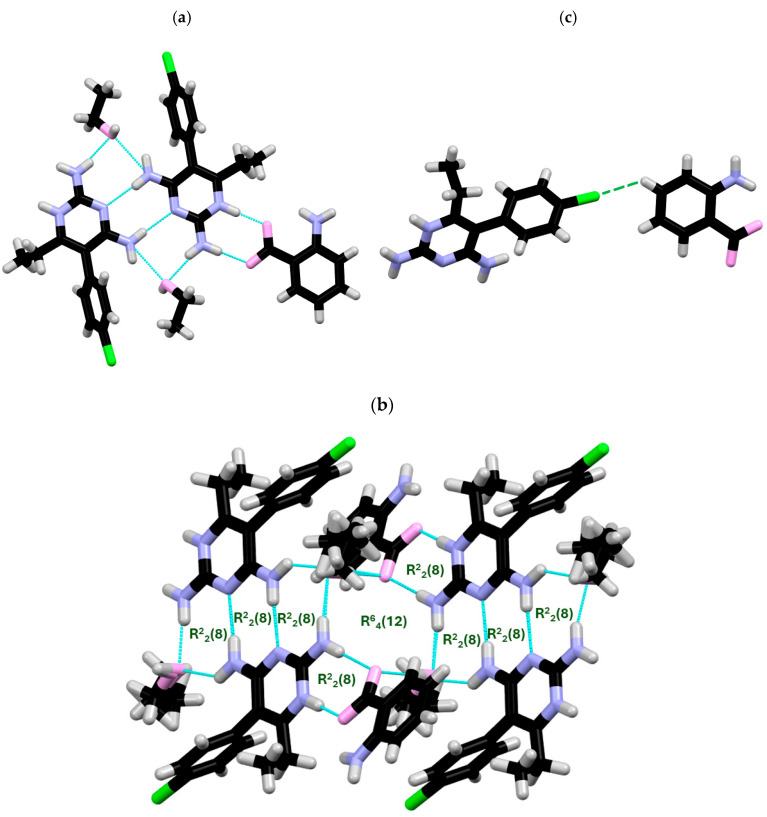
(**a**) H-bonded surroundings of one PYR molecule, (**b**) layout of R^2^_2_(8) and R^6^_4_(12) ring motifs present in the structure, and (**c**) CH⋯Cl interactions between PYR and 3NH_2_-BA.

**X-ray structure of PYR/2NH_2_-BA.** PYR/2NH_2_-BA crystallizes in the triclinic *P*-1 space group, with one protonated at N(1A) PYRH^+^ cation, one 2NH_2_-BA anion, one water molecule disordered over two positions (0.5 occupancy for O1W and 0.5 occupancy for O2W), and one ethanol molecule per asymmetric unit (Figure 2a). The protonated pyrimidine N(1A) atom, and carboxylate O(2B) oxygen atom, amine N(2A)H_2_ group_,_ and O(2B)oxygen atom form an R^2^_2_(8) ring motif via the symmetrical pairing of NH⋯O hydrogen bonds. Another R^2^_2_(8) ring motif is generated via NH⋯N interactions between two symmetrically related PYRH^+^ moieties [N(4)H(4)⋯N(2)]. Amino groups of PYR [N(3A), N(2A)H_2_], as well as the amino group of another PYR moiety [N(4A)H_2_] and the hydroxyl group of ethanol molecules generate consecutive R^2^_2_(8) motifs. A more robust R^6^_4_(12) ring motif, involving a pair of symmetrical interactions of N(2A)H_2,_ carboxylate oxygen O(2B), and a hydroxyl group of ethanol molecules, may also be observed (Figure 2b). The entire structure is additionally stabilized by CH⋯Cl interactions between PYRH^+^ and carboxylate anion (Figure 2c) and CH⋯O interactions between ethanol and water molecules. All of the important interactions are summarized in Table 2.

**Figure 3:**
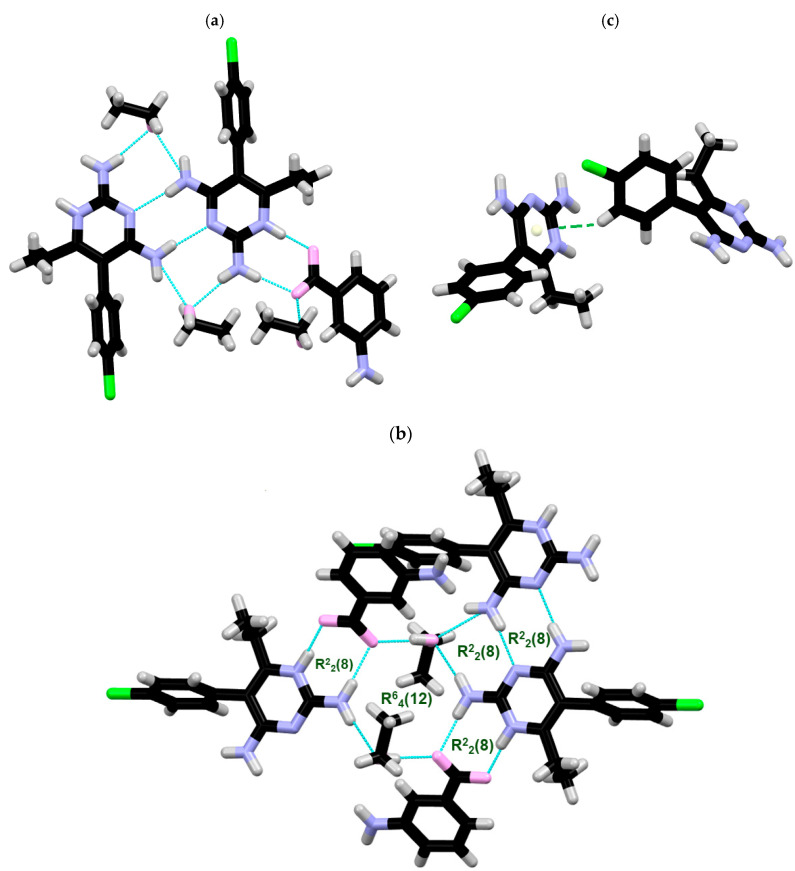
(**a**) H-bonded surroundings of one PYR molecule, (**b**) the layout of R^2^_2_(8) and R^6^_4_(12) ring motifs present in the structure, and (**c**) the CH⋯π interactions two PYR molecules.

**X-ray structure of PYR/3NH_2_-BA.** PYR/3NH_2_-BA crystallizes in the monoclinic *P*2_1_/*n* space group, with one PYR, one 3NH_2_-BA, and one ethanol molecule per asymmetric unit. The similar C−O distances in the carboxylate group of the 3NH_2_-BA moiety (C7B–O1B = 1.256 Å and C7B–O2B = 1.258 Å), as well as the protonation of PYR at N(1) nitrogen atom, prove unambiguously that the obtained associate is a salt. The H-bonded surroundings of the PYR molecule is presented in Figure 3a. The R^2^_2_(8) homosynthon formed by symmetrical N(4A)H(4A)⋯N(3A) hydrogen bonds of two symmetrically related PYR molecules, known from the crystal structures of both of its polymorphs, is sustained in the PYR/3NH_2_-BA salt. Another R^2^_2_(8) motif is formed by the N(4A)H_2_ amino group of one PYRH^+^ cation, an N(3A) nitrogen atom and the N(2)H_2_ amino groups of another PYRH^+^ cation, and an O1E oxygen atom of the ethanol molecule. Additionally, two symmetrical interactions between amino protons at C1A of PYRH^+^ [N(2A)H_2_], the hydroxyl group of an ethanol molecule, and an O2B oxygen atom of a carboxylate anion generate an R^6^_4_(12) ring (Figure 3b). The crystal structure of PYR/3NH_2_-BA is additionally sustained by CH⋯π interactions between the C(11A)H(11A) of one PYRH^+^ and the 2,4-diaminopyrimidine ring of another PYRH^+^ cation (Figure 3c). All of the important interactions are summarized in Table 3.

**Figure 4:**
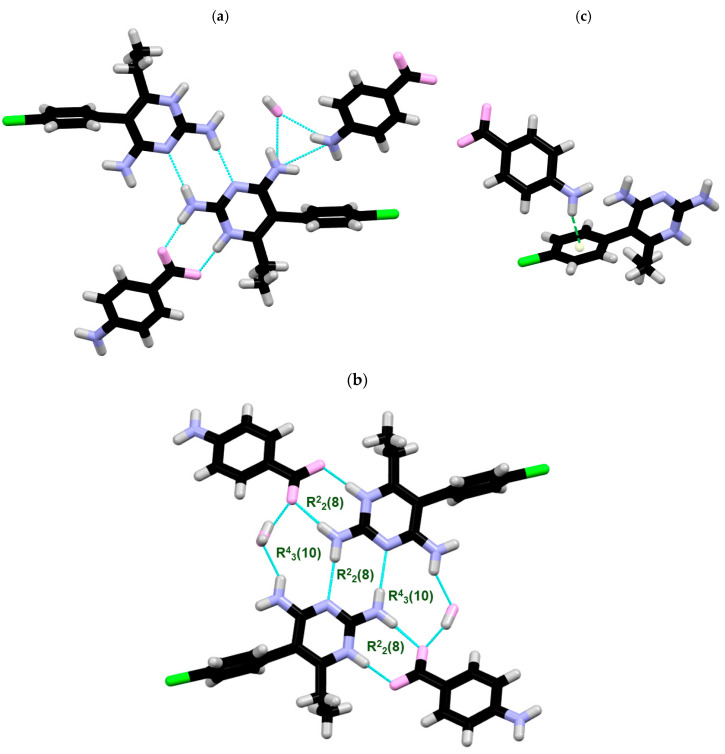
(**a**) H-bonded surroundings of one PYR molecule, (**b**) the layout of the R^2^_2_(8) and R^6^_4_(12) ring motifs present in the structure, and (**c**) the NH⋯π interactions between 4NH_2_-BA and PYR.

**X-ray structure of PYR/4NH_2_-BA.** PYR/4NH_2_-BA crystallizes as a salt in the monoclinic *P*2_1_/*n* space group, with one PYRH^+^ cation, one 3NH_2_-BA anion, and one water molecule per asymmetric unit (Figure 4a). The pyrimidine moieties of two PYRH^+^ cations are linked through a pair of NH⋯N bonds between the N(4A)H2 primary amino group of one PYRH^+^ and the N(3A) nitrogen atom of the second PYRH^+^. The N(1A) nitrogen atom in the pyrimidine ring of the protonated PYRH^+^ is connected with the deprotonated carboxyl group of 4NH_2_-BA, generating an R^2^_2_(8) ring motif. Interactions of two symmetrically related pyrimethamine cations, one carboxylate anion and one water molecule, give rise to an R^4^_3_(10) ring (Figure 4b). The structure is stabilized by NH⋯O interactions between water and PYRH^+^ [N(4A)H(4A1)⋯O1W], as well as with an 3NH_2_-BA anion [N(1B)H(1B2)⋯O1W]. Additionally, the 3D structure is sustained by NH⋯π interactions (Figure 4c). All of the important interactions are summarized in Table 4.

### 2.2. Infrared Spectroscopy (FTIR)

Figure S1 shows the FTIR spectra of PYR, 2NH_2_BA, 3NH_2_B and 4NH_2_B. Each recorded spectrum is consistent with the reference spectra from the NIST Chemistry WebBook and was used for further comparative spectral analysis.

**Figure 5:**
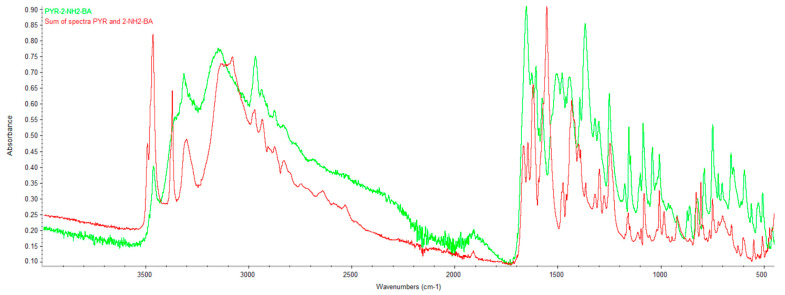
Comparison of the overlaid spectrum of PYR/2NH_2_-BA with the sum of the spectra of PYR and 2NH_2_-BA.

**PYR/2NH_2_-BA.** In the PYR/2NH_2_-BA spectrum (green) (Figure 5 and Figure S2), significant shifts and changes in the band shapes are visible relative to the spectrum obtained by summing the spectra of pure pyrimethamine and 2-aminobenzoic acid (red) (Figure 5 and Figure S2). In the 3600–3200 cm^−1^ range, three distinct bands can be observed for the summed spectra; their shape and intensity differ greatly from the bands in the PYR/2NH_2_-BA spectrum within the same wavenumber range. In the 1700–1400 cm^−1^ region, the PYR/2NH_2_-BA spectrum also shows altered positions and intensities of the bands compared with the sum of the component spectra. In the 1540–1650 cm^−1^ range, the PYR/2NH_2_-BA spectrum exhibits an intense, split band that is not observed in the summed spectra. Similarly, in the 1300–1420 cm^−1^ region, the PYR/2NH_2_-BA spectrum shows a high split band that is absent from the sum of the component spectra. The lack of agreement between the PYR/2NH_2_-BA spectrum and the sum of the spectra of pure PYR and 2NH_2_-BA confirms that the product is not a physical mixture. The observed shifts and changes in the intensity of key bands (characteristic of the –NH and –COOH groups) indicate the formation of new intermolecular interactions.

**Figure 6:**
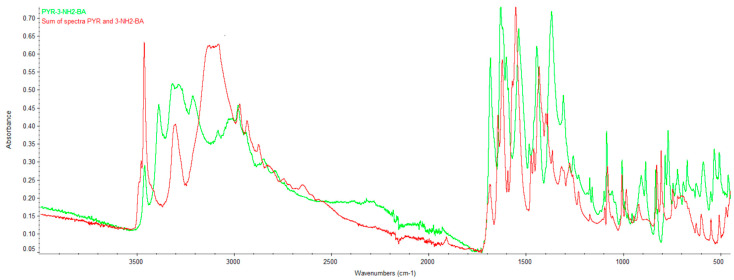
Comparison of the overlaid spectrum of PYR/3NH_2_-BA with the sum of the spectra of PYR and 3NH_2_-BA.

**PYR/3NH_2_-BA.** The 3NH_2_-BA spectrum (green) (Figure S3 and Figure 6) differs from the summed spectra of pyrimethamine and 3-aminobenzoic acid (red) in several regions. In the 3500–3000 cm^−1^ range, changes in band shape and intensity are observed, likely due to amino group vibrations. Shifts and alterations in bands characteristic of the carboxyl group and aromatic ring appear in the 1700–1550 cm^−1^ region. Differences in the 1500–1200 cm^−1^ range further indicate new intermolecular interactions and the formation of a distinct product.

**Figure 7:**
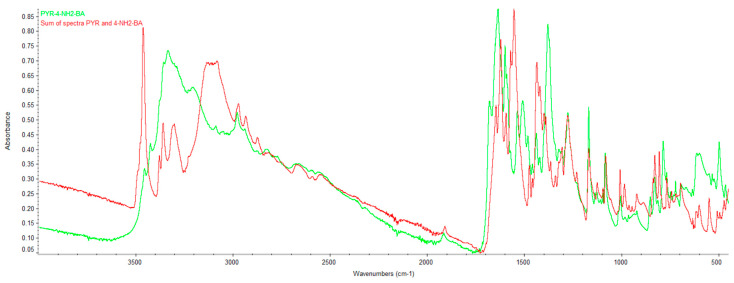
Comparison of the overlaid spectrum of PYR/4NH_2_-BA with the sum of the spectra of PYR and 4NH_2_-BA.

**PYR/4NH_2_-BA.** The 4NH_2_-BA spectrum (green) (Figure 7 and Figure S4) differs, as in the previous cases, from the summed spectra of pyrimethamine and 4-aminobenzoate (red) (Figure 7 and Figure S4) across several wavenumber regions. Once again, in the 3500–3000 cm^−1^ range, significant changes in band shape and intensity are observed (originating from amino group vibrations), which may indicate alterations in hydrogen bonding patterns. In the 1700–1550 cm^−1^ region, shifts and changes in band width are apparent between the PYR/4NH_2_-BA spectrum and the sum of the PYR and 4NH_2_-BA spectra. In the 1500–1200 cm^−1^ range, differences in band intensities and shapes are visible, including the disappearance or splitting of certain signals. The observed shifts and intensity changes in these regions indicate the presence of new intermolecular interactions.

The FTIR analysis of all three studied systems (PYR/2NH_2_-BA, PYR/3NH_2_-BA, and PYR/4NH_2_-BA) shows similar deviations from the sums of the spectra of their individual components. In each case, the product spectra differ from the sum of the spectra of pyrimethamine and the corresponding aminobenzoic acid in several key wavenumber regions. The most significant differences are observed in the 3500–3000 cm^−1^ range, where the band shapes in the product spectra are altered compared with the summed component spectra. Additionally, in the 1700–1550 cm^−1^ region, bands exhibit shifts or changes in shape, and in the 1500–1200 cm^−1^ range, individual signals show marked differences in intensity, with new signals appearing that are absent in the summed spectra or are shifted to different wavenumber values.

### 2.3. Thermal Analysis (DSC/TG)

The substantial change in the thermal behavior of three new solvated salts of PYR tested by simultaneous thermogravimetric analysis (TGA) and differential scanning calorimetry (DSC) compared with the behavior of pre-crystallization compounds indicates the formation of new solid forms. This, in addition to the SC-XRD described before, confirms the formation of PYR/aminobenzoic acids associates.

**Figure 8:**
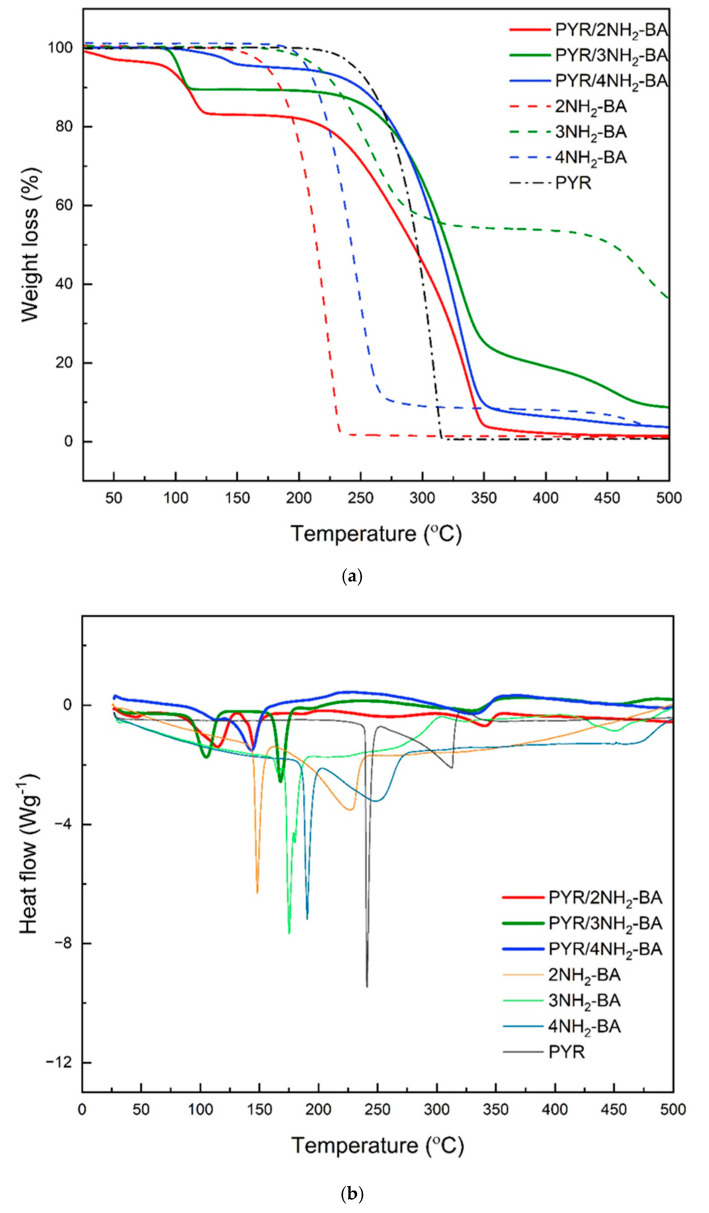
TGA (**a**) and DSC (**b**) curves of PYR salts with aminobenzoic acid isomers and pre-crystallization compounds.

Thermal decomposition of solvated salts of PYR (i.e., PYR/2NH_2_-BA; PYR/3NH_2_-BA, and PYR/4NH_2_-BA) consists of the desolvation stage below 160 °C and two decomposition stages (Figure 8; Table 5). For PYR/2NH_2_-BA and PYR/4NH_2_-BA, the desolvation takes place in two stages, which is consistent with the result of crystallographic data, which revealed the presence of ethanol and water molecules in the structure of these salts. This is also reflected at salt DSC curves through all endothermic peaks below 160 °C related to the solvent release. Above this temperature, salts start to decompose, showing several endothermic peaks at DSC curves. Thermal stability of the aminobenzoic acids isomers is the lowest for 2NH_2_-BA, which starts to decompose at 139.9 °C, followed by 3NH_2_-BA at 162.5 °C, and the most stable is 4NH_2_-BA at 184.5 °C. The aminobenzoic acid solvated salts with PYR start their thermal degradation at lower temperatures, in the same order as the pre-crystallization compounds. The most thermally stable is PYR/4NH_2_-BA/EtOH/H_2_O, which begins to decompose above 170 °C.

### 2.4. Powder X-Ray Diffraction

**Figure 9:**
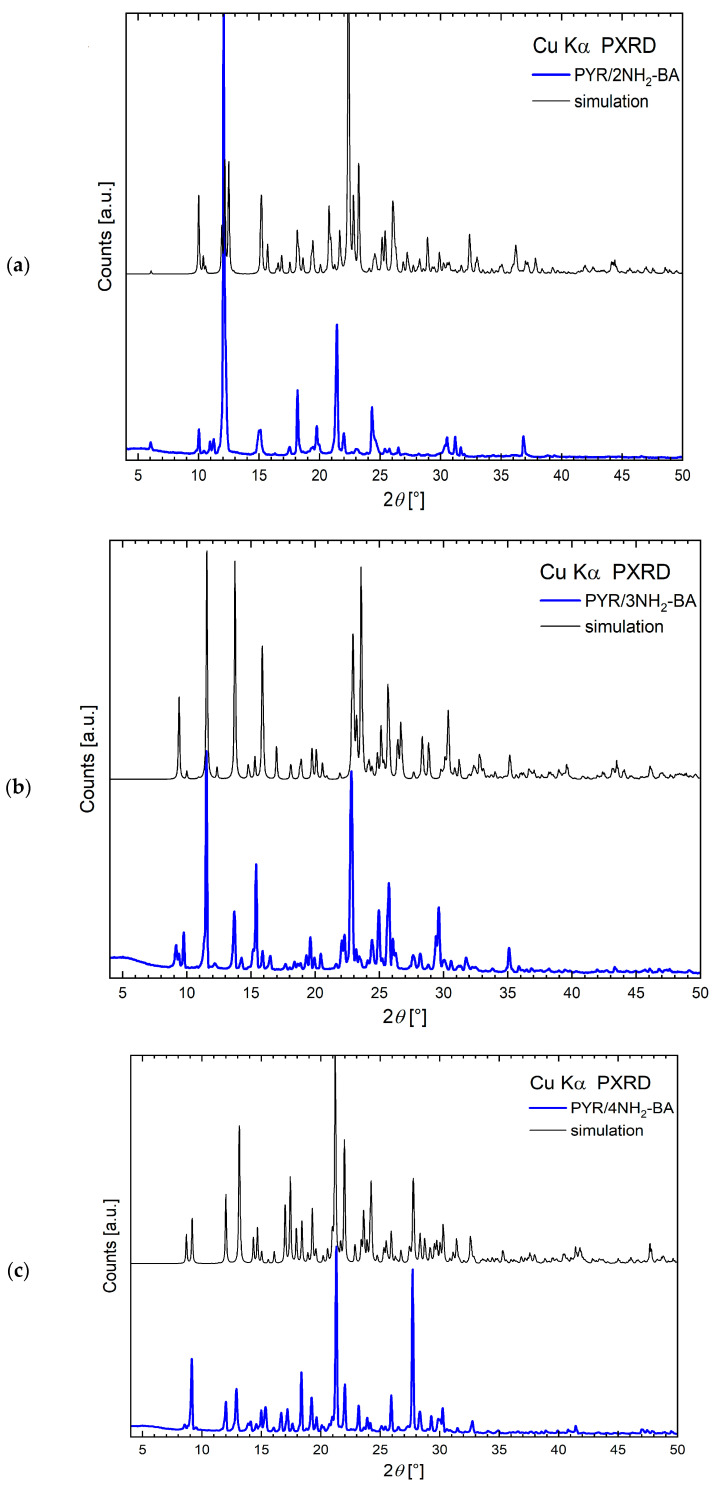
PXRD patterns of solvated PYR salts measured at room temperature (marked in blue) compared with powder X-ray diffraction simulations (marked in black) generated from single crystal X-ray diffraction measurements at 100 K: (**a**) PYR/2NH_2_-BA/EtOH/H_2_O, (**b**) PYR/3NH_2_-BA/EtOH, and (**c**) PYR/4NH_2_-BA/H_2_O.

The measured diffraction patterns are quite similar to those simulated from single crystals (Figure 9). However, some reflections do not align in certain places, which is likely not solely to be due to thermal expansion of the crystal lattice (SCXRD at 100 K, PXRD at room temperature). The differences likely result from the possible presence of phases with different solvent contents in the precipitate, especially since the wet precipitate gradually dried during the measurement.

### 2.5. Mass Spectrometry Measurements

**Figure 10:**
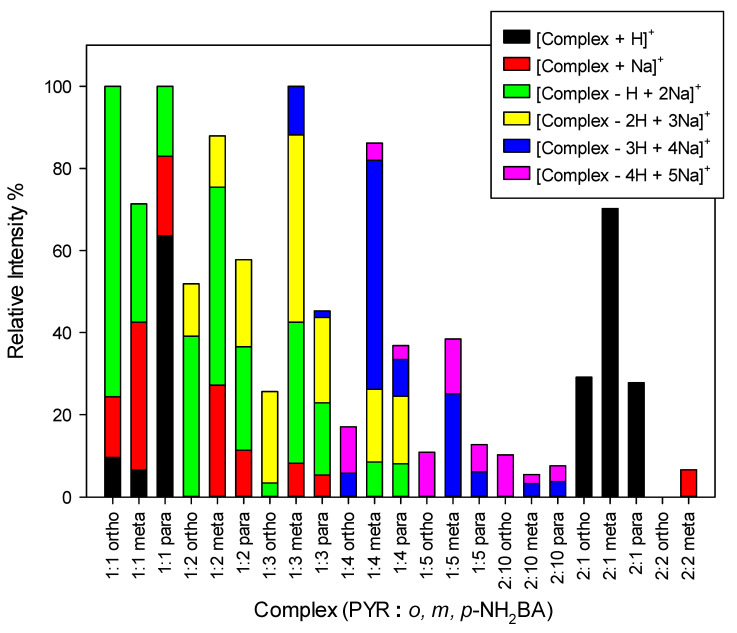
Relative intensity of associates differing in stoichiometry and the types of ions observed in the Q1 mass spectra recorded in positive ion mode (*c* = 0.5 mM).

Mass spectrometry is a well-known method for proving the molecular formula of chemical compounds through high-resolution measurements. By leveraging soft ionization techniques, such as electrospray ionization, this method allows for the observation of peaks corresponding to non-covalently bonded molecules, providing insight into their specific aggregation in solution/gas phase environment [47,48]. The pre-crystallization solutions of PYR and 2NH_2_-BA, 3NH_2_-BA, and 4NH_2_-BA were examined using mass spectrometry. The various types of non-covalent aggregates identified in course of the mass spectra analyses are summarized in Figure 10.

**Figure 11:**
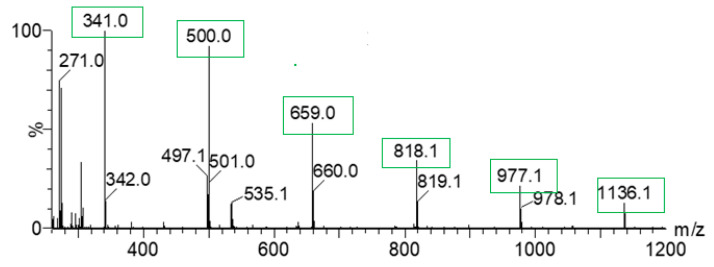
ESI-MS spectrum of 1:1 PYR/3NH_2_BA. Self-associates of NH_2_-BA sodium salt are highlighted in green.

All three isomers of NH_2_BA form non-covalent associates of stoichiometry from 1:1 to 1:5 of PYR to 2NH_2_-BA, 3NH_2_BA, and 4NH_2_-BA. The formation of higher order aggregates is facilitated by the spontaneous self-aggregations of NH_2_-BA—the most intensive peaks in the mass spectra correspond to the formation of sodiated self-associates of NH_2_-BA sodium salt (ions at *m*/*z* = 341, 500, 659, 818, 977, 1136 Da in Figure 11). Additionally, other types of aggregates, such as 2:10, 2:1, and 2:2, were identified. Relative intensities of the particular associates vary with acid isomer.

The associates of 1:1 stoichiometry dominate for 2NH_2_-BA, and 4NH_2_-BA, and higher aggregates reduce as the number of acid units increases. In contrast, higher-order associates of PYR/3NH_2_-BA, 1:3 and 1:4 are more pronounced. The *m*-isomer forms more distinct 2:1 and 2:2 associates compared with the other isomers of NH_2_-BA.

Along with the abundant stoichiometry of PYR/NH_2_-BA associates, they appear as ions of various types and constitutions. These include protonated and sodiated adducts of PYR associates with both NH_2_-BA and NH_2_-BA sodium salt. The associates containing a single PYR molecule are represented by a mixture of different types of ions (different colors of bars in Figure 10), while only protonated ions are observed for 2:1 associates (for details see Figure S5). The preference of an associate to form a particular ion type arises from its different structural and physicochemical properties.

## 3. Materials and Methods

### 3.1. Materials

Pyrimethamine was purchased from Fluorochem (Hadfield, UK) and 2-aminobenzoic acid, 3-aminobenzoic acid, and 4-aminobenzoic acid from TCI (Zwijndrecht, Belgium), each were used without any purification. For crystallization experiments, ethanol and acetone (reagent grade) purchased from POCH (Gliwice, Poland) were used.

### 3.2. Solution Crystallization

All salts were obtained by crystallization from ethanol. Attempts have been made to obtain crystals also from acetone, but in all cases reagents crystallized separately.

**PYR/2NH_2_-BA and PYR/3NH_2_-BA**. A mixture of pyrimethamine (23 mg, 0.09 mmol) with 2-aminobenzoic acid (10.3 mg, 0.08 mmol) and a mixture of pyrimethamine (20.2 mg, 0.081 mmol) with 3-aminobenzoic acid (11.5 mg, 0.084 mmol) were each dissolved in 2 mL of ethanol at 50 °C and cooled to room temperature, yielding crystalline precipitates during solvent evaporation. Filtration afforded colorless plate-like crystals in both cases (10.8 mg, 35% yield for PYR/2NH_2_-BA and 9.62 mg, 30.8% yield for PYR/3NH_2_-BA).

**PYR/4NH_2_-BA**. A ~1:2 mixture of pyrimethamine (20.5 mg, 0.082 mmol) and 4-aminobenzoic acid (23.8 mg, 0.17 mmol) was dissolved in 2 mL of ethanol at 50 °C and then cooled to room temperature, during which crystalline precipitates formed as the solvent evaporated. Filtration afforded colorless, plate-like crystals (23.57 mg, 74.6% yield). Previous attempts to crystallize the product from a 1:1 pyrimethamine–4-aminobenzoic acid mixture in ethanol were unsuccessful, resulting only in separate crystallization of the individual reagents.

### 3.3. Single-Crystal X-Ray Diffraction

The X-ray data were collected on the SuperNova Agilent diffractometer (Rigaku, Tokyo, Japan) using Cu*Kα* radiation (λ = 1.54184 Å). The data were processed with CrysAlisPro, ver. 171.43 [50] The structures were solved using SHELXS [51] and refined using SHELXL2018 [52]. All of the non-hydrogen atoms were refined anisotropically and the hydrogen atoms were placed in the calculated positions. All the graphics were prepared using Mercury, 2022.3.0 [53]. Crystallographic data and the details of refinement are reported in Table 6. Program PLATON [54] was used for analysis of weak interactions within obtained structures. Crystallographic data of the obtained compounds were deposited in the CSD and can be obtained, free of charge, via https://www.ccdc.cam.ac.uk/structures/ from the Cambridge Crystallographic Data Centre, 12, Union Road, Cambridge CB2 1EZ, UK; fax: +44-1223-336033; deposit@ccdc.cam.ac.uk).

### 3.4. Infrared Spectroscopy

All measurements were carried out using a Thermo Scientific Nicolet iS10 FTIR spectrometer with the Attenuated Total Reflectance (ATR) technique. Each sample was analyzed by performing 64 scans at a resolution of 2 cm^−1^. To obtain more accurate results, a background measurement (64 scans at the same resolution) was taken after each analyzed sample. Each solid sample was applied directly onto the diamond crystal of the ATR accessory. A small amount of ethanol was then added to ensure uniform contact between the material and the crystal surface. After the solvent had evaporated, FTIR spectra were recorded.

### 3.5. Thermal Analysis

The simultaneous thermogravimetric analysis (TGA) and differential scanning calorimetry (DSC) measurements were performed using the TGA/DSC 3+ Mettler Toledo device. Before measurements, the crystals obtained after filtration were air-dried for two hours. The 2–3 mg samples of studied compounds were placed in an aluminum crucible and heated up to 500 °C with the 5° min^−1^ rate. All measurements were performed under nitrogen atmosphere.

### 3.6. Powder X-Ray Diffraction

All PXRD measurements were performed at room temperature on an Empyrean Series 2 X-Ray Diffraction System diffractometer, using Cu K*a* radiation. Diffraction patterns were collected as a sum of 4 scans, using Bragg–Brentano θ-θ configuration, over a 2θ range of 4–50° at a scan rate of 2.97° min^−1^, and using zero background holder in a rotating spinner.

### 3.7. Mass Spectrometry

MS measurements were performed on a commercially available quadrupole traveling-wave ion mobility time-of-flight spectrometer (Synapt G2-S HDMS, Waters). Mixtures of 1:1 or 1:2 PYR and 2NH_2_-BA, 3NH_2_-BA and 4NH_2_-BA (c = 0.25 and 0.5 mM) in EtOH were infused through a standard electrospray ion source into the instrument at a flow rate of 10 μL/min. The samples were analyzed in the positive ion mode with a capillary voltage at 3 kV and source temperature at 303 K. The ion products were identified in the mass spectra based on their *m*/*z* values and the accordance between theoretical and experimental ion’s profiles. Selected spectra have been shown in Supporting Information.

## 4. Conclusions

In this work, three new solvated (PYR/2NH_2_-BA/EtOH/H_2_O; PYR/3NH_2_-BA/EtOH, and PYR/4NH_2_-BA/EtOH/H_2_O) salts of antifolate pyrimethamine with isomeric aminobenzoic acids (2NH_2_BA, 3NH_2_BA, and 4NH_2_BA) were obtained by solution crystallization. The salt formation was confirmed by SC XRD, while the purity of the bulk was established by PXRD and FT IR. In all obtained structures typical for pyrimethamine hydrogen bonding motif linking, two PYR molecules were observed—the pyrimidine moieties of two PYRH^+^ cations are linked through a pair of NH⋯N bonds between the N(4A)H_2_ primary amino group of one PYRH^+^ and the N(3A) nitrogen atom of the second PYRH^+^. The 3D structure of PYR salts is additionally stabilized by interactions with solvent molecules, as well as CH⋯Cl (PYR/2NH_2_-BA), CH⋯π (PYR/3NH_2_-BA), and NH⋯π (PYR/4NH_2_-BA) interactions. ESI-MS mass spectrometry studies of pre-crystallization solutions revealed that all three isomers of NH_2_-BA form non-covalent associates with PYR of stoichiometry from 1:1 to 1:5 (PYR to 2NH_2_-BA, 3NH_2_-BA, and 4NH_2_-BA), and that the formation of higher-order aggregates is facilitated by the spontaneous self-aggregations of NH_2_-BA. Overall, this work highlights the crucial role of structural isomerism of aminobenzoic acid coformers in directing supramolecular organization of pyrimethamine salts.

## Figures and Tables

**Table 1:** Distribution of C–O bond lengths for PYR multicomponent crystals.

	D_C(7)–O(1)_ (Å)	D_C(7)–O(2)_ (Å)	Δ*D*_C–O_ (Å)	Proton Transfer
PYR/2NH_2_-BA	1.253 (2)	1.270 (2)	0.017	yes
PYR/3NH_2_-BA	1.256 (2)	1.258 (2)	0.002	yes
PYR/4NH_2_-BA	1.260 (2)	1.268 (2)	0.008	yes

**Table 2:** Geometrical parameters of the intermolecular hydrogen bonds of PYR/2NH_2_-BA.

	D—H⋯A	d(D—H) Å	d(H⋯A) Å	d(D⋯A) Å	<(D—H⋯A) ^o^
1.	O1–H1S⋯O1B ^(a)^	0.82	2.00	2.811 (7)	171
2.	N2A–H2A1⋯O1S ^(b)^	0.86	2.00	2.846 (6)	168
3.	N2A–H2A1⋯O1S’ ^(b)^	0.86	2.19	3.03 (1)	165
4.	N2A–H2A2⋯O1B9 ^(c)^	0.86	1.94	2.790 (4)	171
5.	N4A–H4A1⋯N3A ^(d)^	0.86	2.16	3.015 (3)	175
6.	N4A–H4A2⋯O1S ^(e)^	0.86	2.18	2.844 (7)	134
7.	N4A–H4A2⋯O1S’ ^(e)^	0.86	2.27	2.96 (1)	138
8.	N1B–H1B2⋯O2B	0.86	2.05	2.682 (3)	130
9.	C1S–H1S2⋯O2W	0.97	1.97	2.825 (9)	145
10.	C4B–H4A⋯Cl1 ^(a)^	0.93	2.75	3.466 (3)	135
11.	C12A–H12A⋯O2W ^(d)^	0.93	2.58	3.499 (6)	172

Symmetry operations: ^(a)^ x, −1 + y, z; ^(b)^ 1 − x, −y, 1 − z; ^(c)^ 1 + x, −1 + y, z; ^(d)^ 1 − x, 1 − y, 1 − z; ^(e)^ x, 1 + y, z.

**Table 3:** Geometrical parameters of the intermolecular hydrogen bonds of PYR/3NH_2_-BA.

	D—H⋯A	d(D—H) Å	d(H⋯A) Å	d(D⋯A) Å	<(D—H⋯A) ^o^
1.	O1E–H1E⋯O2B	0.84	1.84	2.665 (2)	167
2.	N1A–H1W⋯O1B ^(a)^	0.91 (2)	1.72 (2)	2.619 (2)	168 (2)
3.	N1B–H1X⋯O2B ^(b)^	0.98 (3)	2.59 (3)	3.563 (2)	174 (2)
4.	N2A–H2X⋯O2B ^(a)^	0.88 (2)	2.01 (2)	2.881 (2)	174 (2)
5.	N2A–H2Z⋯O1E ^(c)^	0.87 (2)	2.11 (2)	2.963 (2)	168 (2)
6.	N4A–H4X⋯O1E	0.88 (2)	2.09 (2)	2.833 (2)	142 (2)
7.	N4A–H4Z⋯N3A ^(c)^	0.90 (2)	2.08 (2)	2.971 (2)	171 (2)
8.	C11A–H11A⋯Cg ^(a)^ ^(b)^	0.95	2.985	3.819	147

Symmetry operations: ^(a)^ 1 + x, y, z ^(b)^ ½ + x, ½ − y, ½ + z ^(c)^ 1 − x, 1 − y, 1 − z.

**Table 4:** Geometrical parameters of the intermolecular hydrogen bonds of PYR/4NH_2_-BA.

	D—H ⋯A	d(D—H) Å	d(H⋯A) Å	d(D⋯A) Å	<(D—H⋯A) ^o^
1.	N1A–H1N⋯O2B ^(a)^	0.94 (2)	1.81 (2)	2.738 (2)	171 (2)
2.	O1W–H1V⋯O1B ^(b)^	0.86 (3)	1.83 (3)	2.662 (2)	164 (2)
3.	O1W–H1W⋯O2B	0.90 (3)	1.88 (3)	2.730 (2)	156 (3)
4.	N2A–H2Y⋯N3A ^(c)^	0.88	2.21	3.084 (2)	173
5.	N2A–H2X⋯O1B ^(a)^	0.88	1.88	2.761 (2)	174
6.	N4A–H4Y⋯O1W	0.88	2.11	2.818 (2)	137
7.	N4A–H4X⋯N1B ^(d)^	0.88	2.33	3.039 (2)	138
8.	N1B–H1R⋯O1W ^(a)^	0.88	2.23	3.069 (2)	158
9.	N1B–H1R⋯N4A ^(a)^	0.88	2.43	3.039 (2)	127
10.	C9B–H9A⋯O1B ^(e)^	0.95	2.59	3.340 (2)	137
11.	C11A–H11A⋯N4A ^(f)^	0.95	2.55	3.579 (2)	164
12	N1B(H1P)⋯Cg ^(a)^) ^(g)^	0.88	3.086	3.722	131

Symmetry operations: ^(a)^ 1 − x, ½ + y, −z ^(b)^ x, ½ − y, −½ + z ^(c)^ 1 − x, 1 − y, −z ^(d)^ 1 − x, ½ + y, ½ − z, ^(e)^ 1 + x, y, z ^(f)^ x, ½ − y, ½ + z ^(g)^ x, 1 + y, z.

**Table 5:** A summary of the weight loss events of PYR, aminobenzoic acids isomers and their salts.

TGA Curves	Desolvation Step (up to 160 °C) Weight (%)		Salt Decomposition Weight (%)	
Substrates				
PYR	-		99.5	
2NH_2_-BA	-		99.5	
3NH_2_-BA	-		46.2	
4NH_2_-BA	-		92.8	
Salts				
PYR/2NH_2_-BA	2.4	13.7	31.2	50.5
PYR/3NH_2_-BA	10.7		69.1	12.4
PYR/4NH_2_-BA	1.1	3.5	88.6	3.8

**Table 6:** Crystallographic data for the studied compounds.

	PYR/2NH_2_-BA	PYR/3NH_2_-BA	PYR/4NH_2_-BA
CCDC	2271560	2271562	2271563
Chem formula	C_12_H_14_N_4_Cl × C_7_H_6_NO_2_ × C_2_H_6_O × H_2_O	C_12_H_14_N_4_Cl × C_7_H_6_NO_2_ × C_2_H_6_O	C_12_H_14_N_4_Cl × C_7_H_6_NO_2_ × H_2_O
Formula Wt	449.93	431.92	403.86
Cryst syst	triclinic	monoclinic	monoclinic
Space group	*P*-1	*P*2_1_/*n*	*P*2_1_/*c*
*a* (Å)	8.7493 (6)	11.9619 (2)	9.7247 (3)
*b* (Å)	9.5143 (7)	15.3108 (3)	15.5035 (5)
c (Å)	15.110 (1)	12.0383 (2)	13.6052 (4)
α (^o^)	74.650 (6)		
β (^o^)	82.736 (6)	94.977 (2)	97.473 (3)
γ (^o^)	73.208 (6)		
V (Å^3^)	1160.3 (2)	2196.46 (7)	2033.79 (11)
Z	2	4	4
D (g × cm^−3^)	1.288	1.306	1.319
T (K)	100	100	100
μ (mm^−1^)	1.763	1.807	1.916
No. of reflections measured	10932	8478	7675
No. of independent reflections	4380	4100	3775
*R*_int_	0.023	0.021	0.02
Final R indices (I > 2s(I))	*R* = 0.0646 w*R* = 0.1780	*R* = 0.036 w*R* = 0.092	*R* = 0.036 w*R* = 0.094
Final R indices (all data)	*R* = 0.0686 w*R* = 0.1822	*R* = 0.042 w*R* = 0.097	*R* = 0.040 w*R* = 0.097
GOF	1.052	1.035	1.027

## Data Availability

All data supporting the findings of this study are available within the article and its Electronic Supplementary Information (ESI). Experimental procedures, full characterization data for all new compounds (SC XRD, IR, PXRD, DSC/TG), and results are provided in the manuscript. Crystallographic data of the new compounds was deposited in the CSD.

## Supplementary Materials

The following supporting information can be downloaded at https://www.mdpi.com/article/10.3390/ijms27010180/s1.

## References

[1] Heppler L.N., Attarha S., Persaud R., Brown J.I., Wang P., Petrova B., Tošić I., Burton F.B., Flamand Y., Walker S.R. (2022). The antimicrobial drug pyrimethamine inhibits STAT3 transcriptional activity by targeting the enzyme dihydrofolate reductase. J. Biol. Chem..

[2] Anderson A.C. (2005). Targeting DHFR in parasitic protozoa. Drug Discov. Today.

[3] Kompis I.M., Islam K., Then R.L. (2005). DNA and RNA Synthesis: Antifolates. Chem. Rev..

[4] Nzila A. (2006). The past, present and future of antifolates in the treatment of Plasmodium falciparum infection. J. Antimicrob. Chemotherap..

[5] Liu H., Qin Y., Zhai D., Zhang Q., Gu J., Tang Y., Yang J., Li K., Yang L., Chen S. (2019). Antimalarial Drug Pyrimethamine Plays a Dual Role in Antitumor Proliferation and Metastasis through Targeting DHFR and TP. Mol. Cancer Ther..

[6] Brown J.I., Persaud R., Iliev P., Karmacharya U., Attarha S., Sahile H., Olsen J.E., Hanke D., Idowu T., Frank D.A. (2024). Investigating the anti-cancer potential of pyrimethamine analogues through a modern chemical biology lens. Eur. J. Med. Chem..

[7] Tommasino C., Gambardella L., Buoncervello M., Griffin R.J., Golding B.T., Alberton M., Macchi D., Spada M., Cerbelli B., d’Amati G. (2016). New derivatives of the antimalarial drug Pyrimethamine in the control of melanoma tumor growth: An in vitro and in vivo study. J. Exp. Clin. Cancer Res..

[8] Cheuka P.M., Njaria P., Mayoka G., Funjika E. (2024). Emerging Drug Targets for Antimalarial Drug Discovery: Validation and Insights into Molecular Mechanisms of Function. J. Med. Chem..

[9] Zhu Z., Chen C., Zhang J., Lai F., Feng J., Wu G., Xia J., Zhang W., Han Z., Zhang C. (2023). Exploration and Biological Evaluation of 1,3-Diamino-7H-pyrrol[3,2-f]quinazoline Derivatives as Dihydrofolate Reductase Inhibitors. J. Med. Chem..

[10] Aitipamula S., Banerjee R., Bansal A.K., Biradha K., Cheney M.L., Roy Choudhury A., Desiraju G.R., Dikundwar A.G., Dubey R., Duggirala N. (2012). Polymorphs, Salts, and Cocrystals: What’s in a Name?. Cryst. Growth Des..

[11] Berry D.J., Steed J.W. (2017). Pharmaceutical cocrystals, salts and multicomponent systems; intermolecular interactions and property based design. Adv. Drug Delivery Rev..

[12] Domingos S., André V., Quaresma S., Martins I.C.B., Minas da Piedade M.F., Duarte M.T. (2015). New forms of old drugs: Improving without changing. J. Pharm. Pharmacol..

[13] Stanley N., Sethuraman V., Muthiah P.T., Luger P., Weber M. (2002). Crystal Engineering of Organic Salts:  Hydrogen-Bonded Supramolecular Motifs in Pyrimethamine Hydrogen Glutarate and Pyrimethamine Formate. Cryst. Growth Des..

[14] Sethuraman V., Stanley N., Muthiah P.T., Sheldrick W.S., Winter M., Luger P., Weber M. (2003). Isomorphism and Crystal Engineering:  Organic Ionic Ladders Formed by Supramolecular Motifs in Pyrimethamine Salts. Cryst. Growth Des..

[15] Delori A., Galek P.T.A., Pidcock E., Jones W. (2012). Quantifying Homo- and Heteromolecular Hydrogen Bonds as a Guide for Adduct Formation. Chem. Eur. J..

[16] Devi P., Muthiah P.T., Row T.N.G., Thiruvenkatam V. (2007). Hydrogen bonding in pyrimethamine hydrogen adipate. Acta Cryst..

[17] Balasubramani K., Muthiah P.T. (2008). Hydrogen-bonding Patterns in Pyrimethaminium Picolinate. Anal. Sci..

[18] Balasubramani K., Muthiah P.T., Bocelli G., Cantoni A. (2007). Pyrimethaminium nicotinate monohydrate. Acta Cryst..

[19] Faroque M.U., Mehmood A., Noureen S., Ahmed M. (2020). Crystal engineering and electrostatic properties of co-crystals of pyrimethamine with benzoic acid and gallic acid. J. Mol. Struc..

[20] Ceborska M., Kędra-Królik K., Narodowiec J., Dąbrowa K. (2021). Influence of Hydroxyl Group Position and Substitution Pattern of Hydroxybenzoic Acid on the Formation of Molecular Salts with the Antifolate Pyrimethamine. Cryst. Growth Des..

[21] O’Malley C., Bouchet C., Manyara G., Walsh N., McArdle P., Erxleben A. (2021). Salts, Binary and Ternary Cocrystals of Pyrimethamine: Mechanosynthesis, Solution Crystallization, and Crystallization from the Gas Phase. Cryst. Growth Des..

[22] Delori A., Galek P.T.A., Pidcock E., Patniac M., Jones W. (2013). Knowledge-based hydrogen bond prediction and the synthesis of salts and cocrystals of the anti-malarial drug pyrimethamine with various drug and GRAS molecules. CrystEngComm.

[23] Darious S.R., Muthiah P.T., Perdih F. (2018). Supramolecular hydrogen-bonding patterns in salts of the antifolate drugs trimethoprim and pyrimethamine. Acta Cryst..

[24] Stanley N., Muthiah P.T., Geib S.J., Luger P., Weber M., Messerschmidt M. (2005). The novel hydrogen bonding motifs and supramolecular patterns in 2,4-diaminopyrimidine–nitrobenzoate complexes. Tetrahedron.

[25] Thanigaimani K., Muthiah P.T., Lynch D.E. (2008). Hydrogen-bonding patterns in the cocrystal 2-amino-4,6-dimethoxypyrimidine–anthranilic acid (1/1). Acta Cryst..

[26] Arman H.D., Kaulgud T., Miller T., Tiekink E.R.T. (2012). Persistence of the {…HOCO…HCN} heterosynthon in the co-crystals formed between anthranilic acid and three bipyridine-containing molecules. Z. Krist..

[27] Madusanka N., Eddleston M.D., Arhangelskis M., Jones M. (2014). Polymorphs, hydrates and solvates of a co-crystal of caffeine with anthranilic acid. Acta Cryst..

[28] Fischer F., Joester M., Rademann K., Emmerling F. (2015). Survival of the Fittest: Competitive Co-crystal Reactions in the Ball Mill. Chem. Eur. J..

[29] Djaló M., Cunha A.E.S., Luís J.P., Quaresma S., Fernandes A., André V., Duarte M.T. (2021). Sparfloxacin Multicomponent Crystals: Targeting the Solubility of Problematic Antibiotics. Cryst. Growth Des..

[30] Dai X.-L., Li S., Chen J.-M., Lu T.-B. (2016). Improving the Membrane Permeability of 5-Fluorouracil via Cocrystallization. Cryst. Growth Des..

[31] Vangala V.R., Chow P.S., Tan R.B.H. (2012). Co-Crystals and Co-Crystal Hydrates of the Antibiotic Nitrofurantoin: Structural Studies and Physicochemical Properties. Cryst. Growth Des..

[32] Singh M., Anthal S., Srijana P.J., Narayana B., Sarojini B., Likhitha K., Kamal U., Kant R. (2022). Novel supramolecular co-crystal of 3-aminobenzoic acid with 4-acetyl-pyridine: Synthesis, X-ray structure, DFT and Hirshfeld surface analysis. J. Mol. Struc..

[33] Xie Y., Gong L., Tao Y., Zhang B., Zhang L., Yang S., Yang D., Lu Y., Du G. (2024). New Cocrystals of Ligustrazine: Enhancing Hygroscopicity and Stability. Molecules.

[34] Shukla A., Khan E., Srivastava K., Sinha K., Tandon P., Vangala V.R. (2017). Study of molecular interactions and chemical reactivity of the nitrofurantoin–3-aminobenzoic acid cocrystal using quantum chemical and spectroscopic (IR, Raman, ^13^C SS-NMR) approaches. CrystEngComm.

[35] Harriss B.I., Vella-Zarb L., Wilson C., Radosavljevic Evans I. (2013). Furosemide Cocrystals: Structures, Hydrogen Bonding, and Implications. Cryst. Growth Des..

[36] Maddileti D., Jayabun S.K., Nangia A. (2013). Soluble Cocrystals of the Xanthine Oxidase Inhibitor Febuxostat. Cryst. Growth Des..

[37] Bhandaru J.S., Malothu N., Akkinepally R.R. (2015). Characterization and Solubility Studies of Pharmaceutical Cocrystals of Eprosartan Mesylate. Cryst. Growth Des..

[38] Suresh K., Minkov V.S., Kumar Namila K., Derevyannikova E., Losev E., Nangia A., Boldyreva E.V. (2015). Novel Synthons in Sulfamethizole Cocrystals: Structure–Property Relations and Solubility. Cryst. Growth Des..

[39] Fernandes J.A., Sardo M., Mafra L., Choquesillo-Lazarte D., Masciocchi N. (2015). X-Ray and NMR Crystallography Studies of Novel Theophylline Cocrystals Prepared by Liquid Assisted Grinding. Cryst. Growth Des..

[40] Singaraju A.B., Nguyen K., Gawędzki P., Herald F., Meyer G., Wentworth D., Swenson D.C., Stevens L.L. (2017). Combining Crystal Structure and Interaction Topology for Interpreting Functional Molecular Solids: A Study of Theophylline Cocrystals. Cryst. Growth Des..

[41] Pan X., Zheng Y., Chen R., Qiu S., Chen Z., Rao W., Chen S., You Y., Lü J., Xu L. (2019). Cocrystal of Sulfamethazine and p-Aminobenzoic Acid: Structural Establishment and Enhanced Antibacterial Properties. Cryst. Growth Des..

[42] Fandiño O.E., Reviglio L., Linck Y.G., Monti G.A., Marcos Valdez M.M., Faudone S.N., Caira M.R., Sperandeo N.R. (2020). Novel Cocrystals and Eutectics of the Antiprotozoal Tinidazole: Mechanochemical Synthesis, Cocrystallization, and Characterization. Cryst. Growth Des..

[43] Martin F., Pop M., Kacso I., Grosu I.G., Miclăuş M., Vodnar D., Lung I., Filip G.A., Olteanu E.D., Moldovan R. (2020). Ketoconazole-*p*-aminobenzoic Acid Cocrystal: Revival of an Old Drug by Crystal Engineering. Mol. Pharm..

[44] Deka P., Gogoi D., Althubeiti K., Rao D.R., Thakuria R. (2021). Mechanosynthesis, Characterization, and Physicochemical Property Investigation of a Favipiravir Cocrystal with Theophylline and GRAS Coformers. Cryst. Growth Des..

[45] Hibbard T., Shankland K., Al-Obaidi H. (2024). Preparation and formulation of progesterone *para*-aminobenzoic acid co-crystals with improved dissolution and stability. Europ. J. Pharm. Biopharm..

[46] Xue N., He B., Jia Y., Yang C., Wang J., Li M. (2020). The mechanism of binding with the α-glucosidase in vitro and the evaluation on hypoglycemic effect in vivo: Cocrystals involving synergism of gallic acid and conformer. Europ. J. Pharm. Biopharm..

[47] Chen F., Gülbakan B., Weidmann S., Fagerer S.R., Ibáñez A.J., Zenobi R. (2016). Applying mass spectrometry to study non-covalent biomolecule complexes. Mass Spectrom. Rev..

[48] Casas-Hinestroza J.L., Bueno M., Ibáñez E., Cifuentes A. (2019). Recent advances in mass spectrometry studies of non-covalent complexes of macrocycles—A review. Anal. Chim. Acta.

[49] Allen F.H., Kennard O., Watson D.G., Brammer L., Orpen A.G., Taylor R. (1987). Tables of Bond Lengths determined by X-Ray and Neutron Diffraction. Part I. Bond Lengths in Organic Compounds. J. Chem. Soc. Perkin Trans. 2.

[50] (2020). CrysAlisPRO, *1.171.40.84a*.

[51] Sheldrick G.M. (2008). A short history of SHELX. Acta Cryst. A.

[52] Sheldrick G.M. (2015). Crystal structure refinement with SHELXL. Acta Cryst..

[53] Macrae C.F., Sovago I., Cottrell S.J., Galek P.T.A., McCabe P., Pidcock E., Platings M., Shields G.P., Stevens J.S., Towler M. (2020). *Mercury* 4.0: From visualization to analysis, design and prediction. J. Appl. Cryst..

[54] Spek A.L. (2003). Single-crystal structure validation with the program PLATON. J. Appl. Crystallogr..

