# Supplementary material for: Salts of Antifolate Pyrimethamine with Isomeric Aminobenzoic Acids: Exploring Packing Interactions and Pre-Crystallization Aggregation
Source: Int J Mol Sci. 2025 Dec 23;27(1):180. doi: 10.3390/ijms27010180 (PMC12785875; doi:10.3390/ijms27010180)
Supplement: Supplementary file 1 [file ijms-27-00180-s001.zip › ijms-3947203-supplementary.pdf]

Supporting Information for

Salts of Antifolate Pyrimethamine with isomeric aminobenzoic acids: Exploring Packing Interactions and Pre-Crystallization Aggregation

Karolina Cichocka [1], Magdalena Zimnicka [2], Karolina Kędra [3], Arkadiusz Gajek [3] and Magdalena Ceborska [1,*]

1 Faculty of Mathematics and Natural Sciences, Cardinal Stefan Wyszyński University, 01-938 Warsaw, Poland; karolina.cichocka@student.uksw.edu.pl
2 Institute of Organic Chemistry, Polish Academy of Sciences, 01-224 Warsaw, Poland; magdalena.zimnicka@icho.edu.pl
3 Institute of Physical Chemistry, Polish Academy of Sciences, 01-224 Warsaw, Poland; kkedra@ichf.edu.pl (K.K.); agajek@ichf.edu.pl (A.G.)
* Correspondence: m.ceborska@uksw.edu.pl

1. **Infrared spectroscopy……………………………………………………...……2**
2. **Mass spectrometry…………………………………...……………………3**

1. Infrared spectroscopy

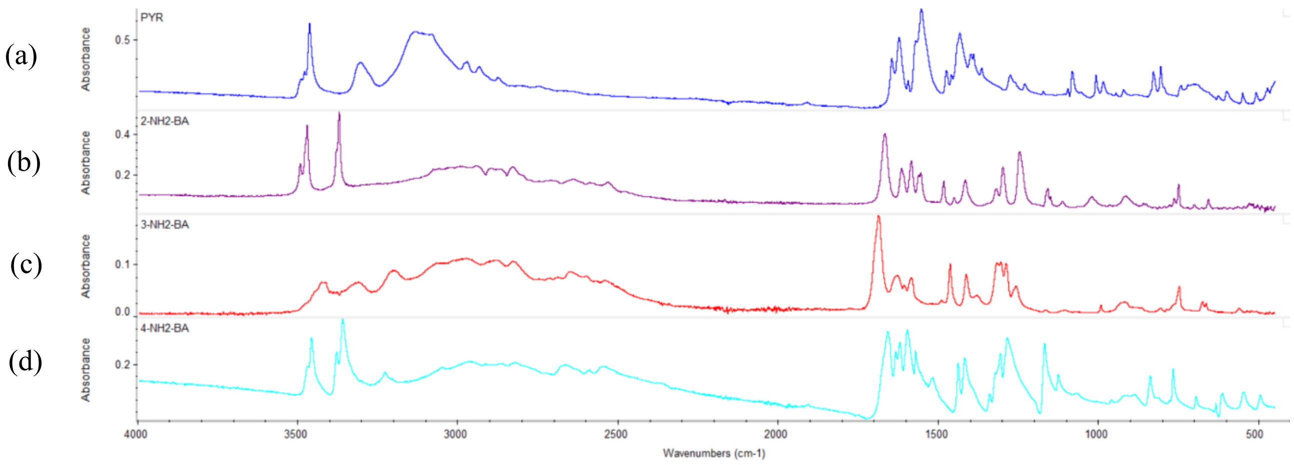

Figure S1. FT-IR spectrum of a) PYR b) $2NH_2$-BA c) $3NH_2$-BA d) $4NH_2$-BA.

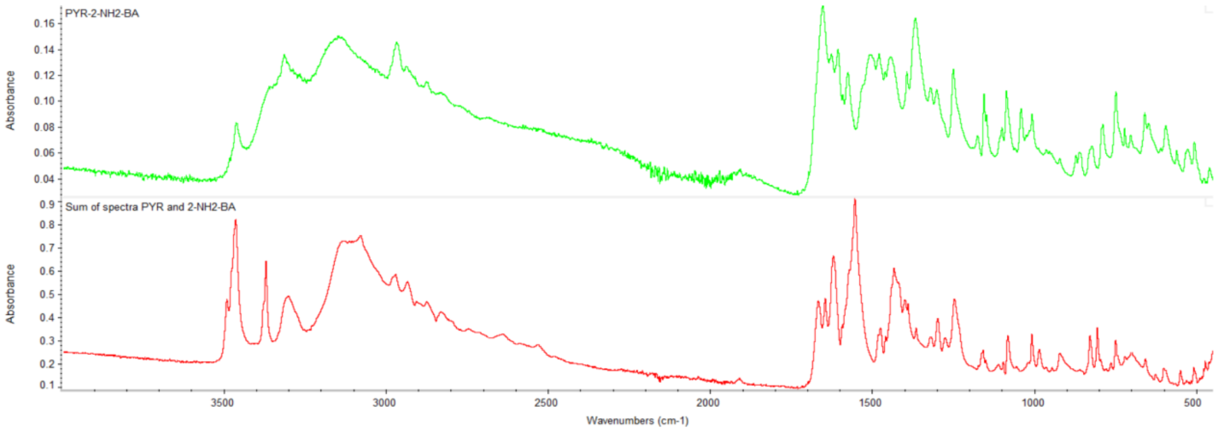

Figure S2. Comparison of the spectrum of PYR/$2NH_2$-BA with the sum of the spectra of PYR and $2NH_2$-BA.

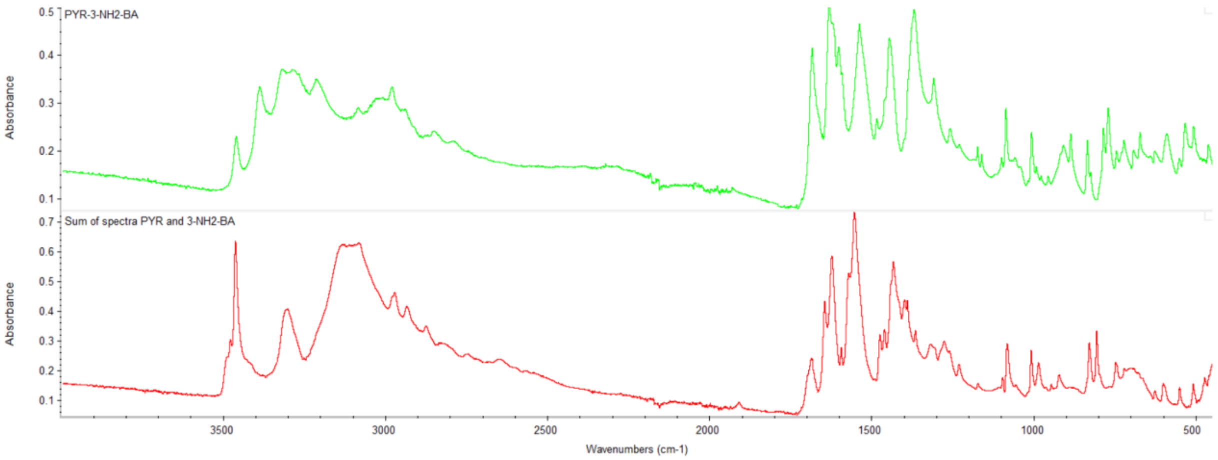

Figure S3. Comparison of the spectrum of PYR/$3NH_2$-BA with the sum of the spectra of PYR and $3NH_2$-BA.

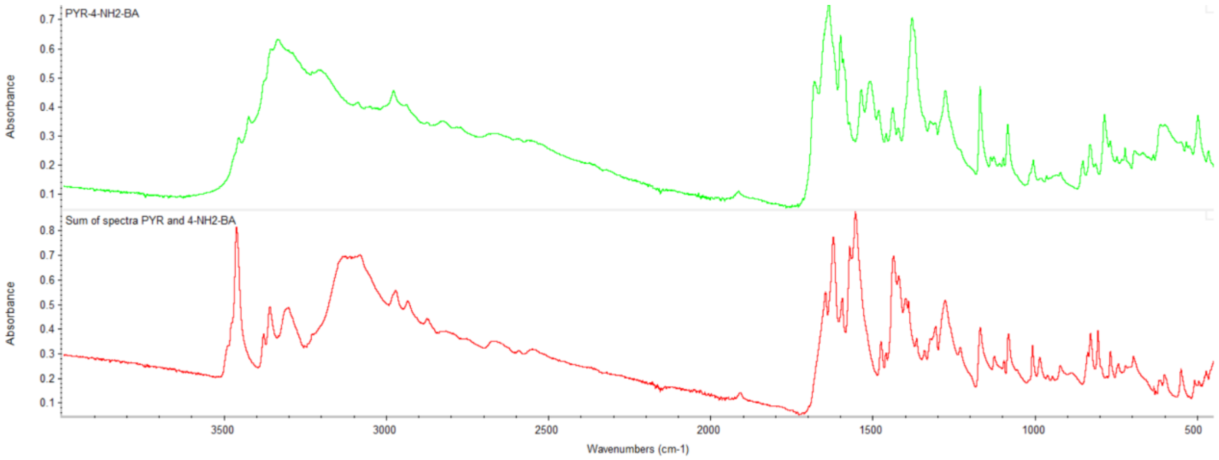

Figure S4. Comparison of the spectrum of PYR/4NH_2-BA with the sum of the spectra of PYR and 4NH_2-BA.

2. **Mass Spectrometry**

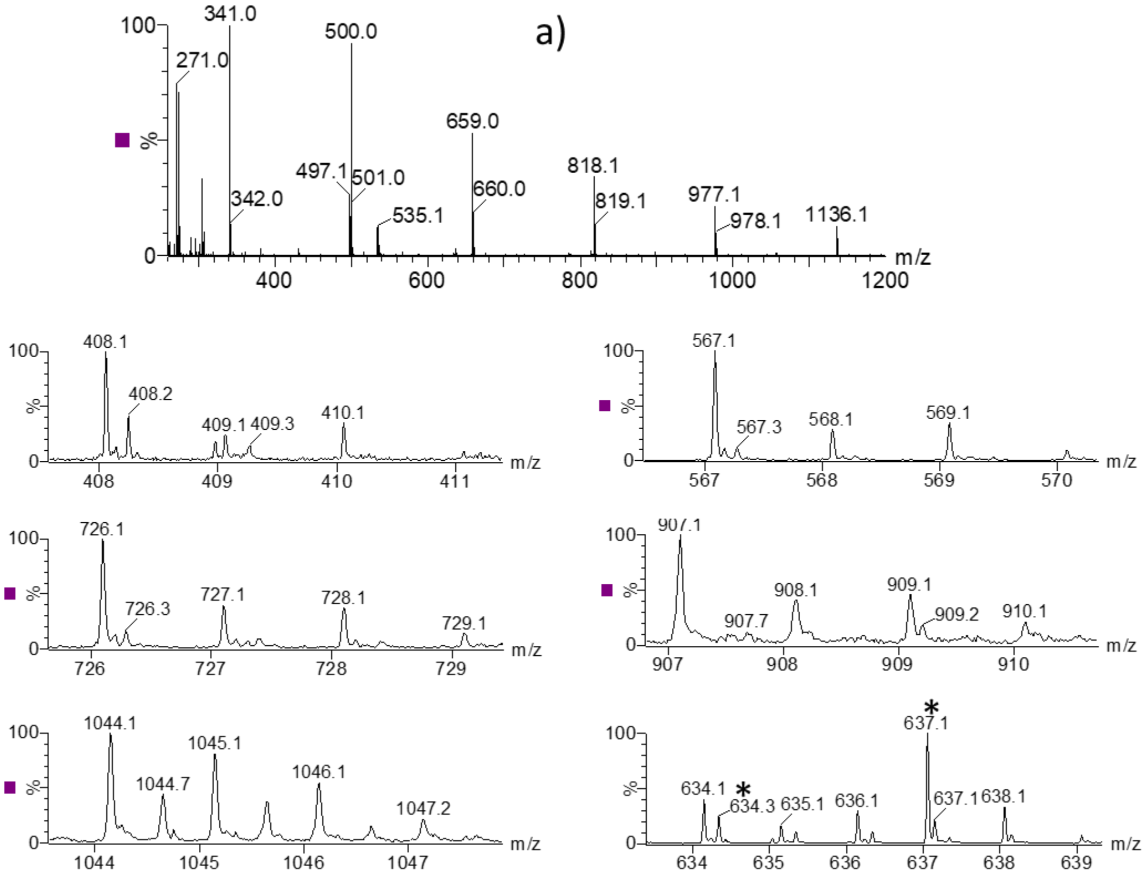

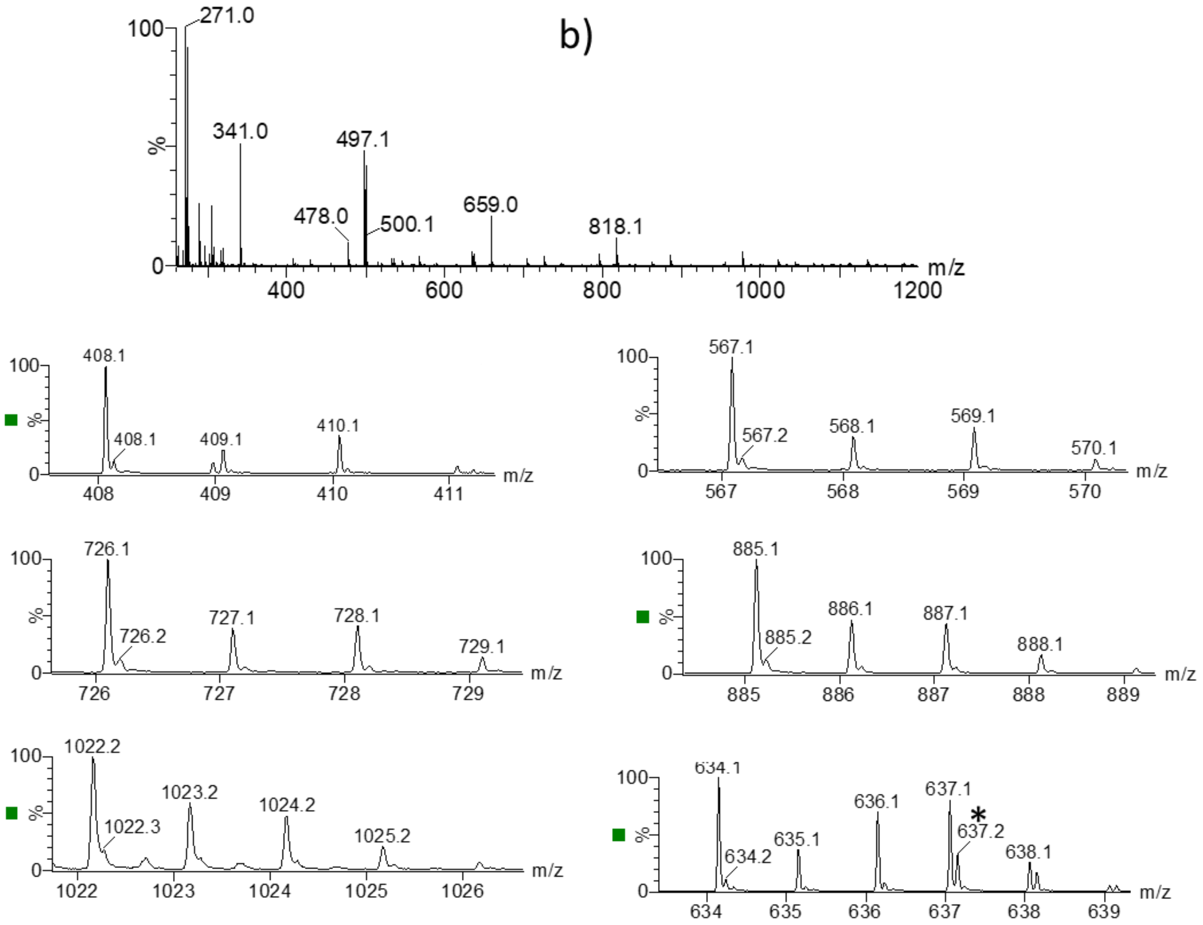
b)
271.0
341.0
497.1
478.0
500.1
659.0
818.1
m/z
408.1
408.1
409.1
410.1
567.1
567.2
568.1
569.1
570.1
726.1
726.2
727.1
728.1
729.1
885.1
885.2
886.1
887.1
888.1
1022.2
1022.3
1023.2
1024.2
1025.2
634.1
634.2
635.1
636.1
637.1
637.2
638.1
*

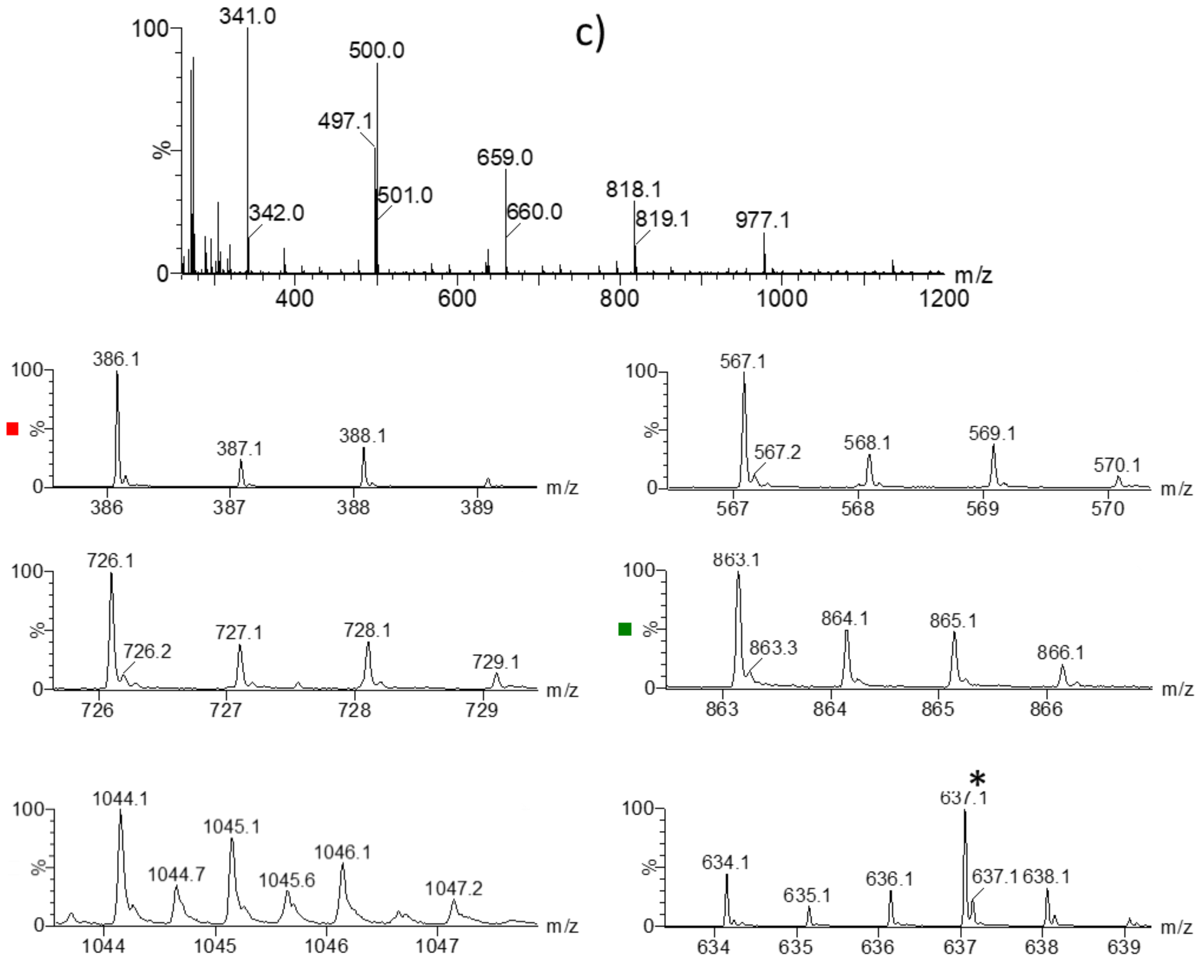

Figure S5. Full and selected ranges of Q1-MS spectra recorded for the mixtures of PYR and a) *o*-NH2BA, b) *m*-NH2BA, and c) *p*-NH2BA in EtOH. The peak assignment is presented in Table S1. *Peaks of impurities.

Table S1. Type of aggregates and their m/z values.

Stoichiometry of PYR : NH_2BA Complex	Ion Type	*m/z*
1:1	$[Complex + H]^+$	386
	$[Complex + Na]^+$	408
	$[Complex - H + 2Na]^+$	430
1:2	$[Complex + H]^+$	523
	$[Complex + Na]^+$	545
	$[Complex - H + 2Na]^+$	567
	$[Complex - 2H + 3Na]^+$	589
1:3	$[Complex + H]^+$	660
	$[Complex + Na]^+$	682
	$[Complex - H + 2Na]^+$	704
	$[Complex - 2H + 3Na]^+$	726
	$[Complex - 3H + 4Na]^+$	748
1:4	$[Complex + H]^+$	797
	$[Complex + Na]^+$	819
	$[Complex - H + 2Na]^+$	841
	$[Complex - 2H + 3Na]^+$	863
	$[Complex - 3H + 4Na]^+$	885
	$[Complex - 4H + 5Na]^+$	907
1:5	$[Complex + H]^+$	934
	$[Complex + Na]^+$	956
	$[Complex - H + 2Na]^+$	978
	$[Complex - 2H + 3Na]^+$	1000
	$[Complex - 3H + 4Na]^+$	1022
	$[Complex - 4H + 5Na]^+$	1044
2:10	$[Complex - 6H + 8Na]^{2+}$	1022
	$[Complex - 8H + 10Na]^{2+}$	1044
2:1	$[Complex + H]^+$	634
